# *In vitro* evaluation of microbial D- and L-lactate production as biomarkers of infection

**DOI:** 10.3389/fmicb.2024.1406350

**Published:** 2024-08-08

**Authors:** Paula Morovic, Mercedes Gonzalez Moreno, Andrej Trampuz, Svetlana Karbysheva

**Affiliations:** Center for Musculoskeletal Surgery (CMSC), Charité – Universitätsmedizin Berlin, Corporate Member of Freie Universität Berlin, Humboldt-Universität zu Berlin and Berlin Institute of Health, Berlin, Germany

**Keywords:** biomarker, infection, D-lactate, joint, biofilm

## Abstract

Mammalian cells produce and metabolize almost exclusively L-lactate, bacterial species have the capacity to produce both D-lactate and L-lactate. The aim of this study was to evaluate the intrinsic production of D- and L-lactate in the most common pathogenic microorganisms causing septic arthritis (SA) and periprosthetic joint infection (PJI) as a potential biomarker for the diagnosis of infection. Following microorganisms were grown according to ATCC culture guides and tested for production of D- and L-lactate: *Staphylococcus aureus* (ATCC 43300), *Staphylococcus epidermidis* (ATCC 35984), *Enterococcus faecalis* (ATCC 19433), *Streptococcus pyogenes* (ATCC 19615), *Escherichia coli* (ATCC 25922), *Pseudomonas aeruginosa* (ATCC 27853), *Cutibacterium acnes* (ATCC 11827), and *Candida albicans* (ATCC 90028). Pathogens were inoculated in 8 ml of appropriate liquid media and incubated as planktonic or biofilm form in either aerobic, anaerobic or CO_2_ atmosphere up to 312 h. D- and L-lactate measurements were performed at different time points: 0, 6, 9, 12 and 24 h, then once per day for slow-growing pathogens. Samples were serially diluted and plated for colony counting. Liquid culture media without microorganisms served as a negative control. Production of D-lactate was observed in all tested microorganisms, whereas no L-lactate was detected in *E. coli*, *P. aeruginosa,* and *C. albicans*. Maximal concentration of D-lactate was produced by *S. aureus* (10.99 mmol/L), followed by *E. coli* (1.22 mmol/L), and *S. epidermidis* (0.48 mmol/L). Maximal L-lactate concentration was observed in *S. pyogenes* (10.12 mmol/L), followed by *S. aureus* (9.71 mmol/L), *E. faecalis* (2.64 mmol/L), and *S. epidermidis* (2.50 mmol/L). *S. epidermidis* bacterial biofilm produced significantly higher amount of D- and L-lactate compared to planktonic form (*p* = 0.015 and *p* = 0.002, respectively). Our study has demonstrated that the most common pathogenic microorganisms causing SA and PJI have the capability to generate measurable amounts of D-lactate in both planktonic and biofilm form, highlighting the practical value of this biomarker as an indicator for bacterial and fungal infections. In contrast to D-lactate, the absence of L-lactate production in certain tested bacteria, as well as in fungi, suggests that L-lactate is not eligible as a biomarker for diagnosing microbial infections.

## Introduction

D- and L-lactate are stereoisomers of lactic acid. Mammalian cells produce and metabolize almost exclusively L-lactate leading to millimolar concentrations in blood plasma (Levitt and Levitt, 2020). D-lactate can be found in human serum only in nanomolar concentrations as a product of methylglyoxal metabolism or gut bacteria (Paoli et al., 2010). Methylglyoxal pathway is the endogenous source of D-lactate in healthy people with the normal plasma concentration from 0.006 to 0.02 mmol/L (Ewaschuk et al., 2005; de Bari et al., 2019; Levitt and Levitt, 2020). One of the exogenous sources of D-lactate is its production by several strains of bacteria in the gastrointestinal tract. The plasma D- and L-lactate can be increased to 0.2 and 2.2 mmol/L, respectively, in patients with intestinal barrier dysfunction due to intestinal ischemia (Assadian et al., 2006; Evennett et al., 2009). In patients with resected or defunctionalized small bowel who develop D-lactate acidosis the concentration of D-lactate can reach 10.0 mmol/L (Smith et al., 1986; Uribarri et al., 1998; Levitt and Levitt, 2020).

In contrast to mammalian cells, bacterial species have the capacity to produce both D-lactate and L-lactate in millimolar concentrations. Several studies have suggested that D-lactate may be of value as a clinical diagnostic tool and potential biomarker of infections such as pleuritis, ascites, meningitis, sepsis, septic arthritis (SA) and periprosthetic joint infections (PJI; Smith et al., 1986; Poeze et al., 2003; Chen et al., 2012; Karbysheva et al., 2020b). Moreover, elimination rate of D-lactate from human body is lower than that of L-lactate, which could make D-lactate test useful for diagnosing infection in patients with initiated antibacterial treatment (McLellan et al., 1992; Pohanka, 2020).

There have been several *in vitro* studies investigating bacterial D- and L-lactate production (Smith et al., 1986; Wellmer et al., 2001; Lefèvre et al., 2023). The main focus of those studies was the assessment of D- and L-lactate in highly virulent pathogens such as *Staphylococcus aureus* and *Escherichia coli* and potential application of these biomarkers for the diagnosis of bacterial meningitis and sepsis (Wellmer et al., 2001; Lefèvre et al., 2023). In contrast, the most common pathogens in bone and joint infections are represented by both high and low virulent pathogens. SA is caused mostly by high virulent bacteria such as *S. aureus* (in 40–60% of the cases), streptococci (20–30%), enterococci (10–20%) and gram-negative bacteria (10–20%; Momodu and Savaliya, 2024). In PJI the low-virulence pathogens are primarily detected, namely coagulase-negative staphylococci in 43–60% of the cases, followed by other microorganisms such as *S. aureus* (12–23%), streptococci and enterococci (both up to 10%). Aerobic gram-negative bacilli are involved in 3 to 6% of the cases. *Cutibacterium acnes* is the most common anaerobe attributed to 10% of PJI, predominantly in shoulder prosthesis infections. Fungi are found in 1–2% of all PJIs, with *Candida* species being predominant (Zimmerli et al., 2004; Tande and Patel, 2014; Chisari et al., 2022). Except for the planktonic form, all these pathogens can form a layer on the surface of the implant and persist as a biofilm in a low metabolism state, making the diagnosis and treatment even more demanding (Trampuz et al., 2007; Lichtenberg et al., 2022).

The goal of this *in vitro* study was to evaluate the intrinsic production of D- and L-lactate in the most common microorganisms causing SA and PJI. Specifically, the study aimed to investigate the levels of D- and L-lactate in planktonic cultures of various pathogens, as well as in a biofilm culture of *Staphylococcus epidermidis*, which serves as a model for chronic low-grade PJI. We believe that this knowledge will provide new insights into D- and L-lactate physiology and usefulness of measuring these enantiomers of lactic acid in the diagnosis of infection.

## Materials and methods

### Bacterial strains and growth conditions

Stocks of *S. aureus* (ATCC 43300), *S. epidermidis* (ATCC 35984), *Enterococcus faecalis* (ATCC 19433), *Streptococcus pyogenes* (ATCC 19615), *E. coli* (ATCC 25922), *Pseudomonas aeruginosa* (ATCC 27853), *C. acnes* (ATCC 11827), and *Candida albicans* (ATCC 90028) were stored in cryovial bead preservation system at −80°C until analysis. The strains were grown according to ATCC culture guides. Staphylococci, *E. coli and P. aeruginosa* were cultured on Trypticase Soy Agar (TSA) and in Trypticase Soy Broth (TSB) at 37°C in an ambient air incubator. *E. faecalis*, streptococci and anaerobes were cultivated on Trypticase Soy Agar supplemented with 5% defibrinated sheep blood (TSA + 5% DSB) and in TSB with 5% defibrinated sheep blood (TSB + 5% DSB) at 37°C under 5% CO_2_ or anaerobic atmosphere. *C. albicans was* cultured in Sabouraud broth and agar at 30°C in an ambient air incubator. After cultivation on solid media for 24 h, inoculum of each bacterial strain equal McFarland 0.5 standard were prepared in 0.9% NaCl. Liquid cultures were prepared in 8 ml of appropriate liquid culture media to a concentration of 1E+01 CFU/ml and incubated in either aerobic, anaerobic or CO_2_ atmosphere. All culture media were purchased from Sigma-Aldrich, St. Louis, MO, USA.

### *Staphylococcus epidermidis* biofilm formation

As a model to form the bacterial biofilm porous glass beads (diameter 4 mm, pore sizes 60 μm, ROBU1, Hattert, Germany) were used. The beads were placed in 2 ml of TSB containing 1E+08 CFU/ml inoculum of *S. epidermidis*. After 24 h, beads were re-incubated in fresh TSB and biofilms were statically grown for further 72 h at 37°C, as previously described (Karbysheva et al., 2020a). After biofilm formation, beads were washed three times with 2 ml 0.9% NaCl to remove planktonic bacteria, transferred into 8 ml of fresh TSB and incubated aerobically and anaerobically at 37°C for 2 weeks. Biofilm supernatant samples (150 μl) were collected for D- and L-lactate analysis and CFU counting.

### D- and L-lactate assays

For all pathogens D- and L-lactate measurements were performed at different time points: immediately after inoculation in liquid media with final concentration of 1E+01 CFU/ml (0 h); after 6, 9, 12, 24, 48, 72, and 144 h, except for *S. epidermidis* and *C. acnes* which were measured for a total of 312 h (14 days). The same procedure was performed with liquid culture media (TSB, TSB + 5% DSB and Sabouraud broth) without bacteria which served as a negative control.

D- and L-lactate measurements were performed using D-lactate and L-lactate colorimetric assay kits (Abcam, Cambridge, UK) according to manufacturer’s instructions. Optical density of the reaction was measured spectrophotometrically and calculated to concentration of lactate in mmol/l, using the standard calibration curve. Before measurement all samples were made protein-free by ultrafiltering 300 μl of liquid culture using 10 kDa filter at 4°C for 60 min. (Microcon-10 kDa Centrifugal Filter Unit with Ultracel-10 membrane, Milipore, Merck KGaA, Darmstadt, Germany). Final concentrations of microbial D- and L-lactate was calculated by subtracting the negative control from the D- and L-lactate measurements in bacterial cultures.

### Colony counting (CFU) method

In parallel with lactate measurements, all samples including negative controls were serially diluted and plated on TSA for colony counting. For all bacterial strains the growth curves were created.

### Effect of the glucose on D- and L-lactate production

In a separate planktonic culture of *S. aureus* glucose was added to final concentration of 5% after 24 and 48 h of incubation. The sample preparation procedure, growth conditions, D- and L-lactate measurement, as well as colony counting was performed as described above.

All measurements were performed in triplicates, on three different days.

### Statistical methods

SigmaPlot (version 13.0; Systat Software, Chicago, IL, USA) and Prism (version 8; GraphPad, La Jolla, CA, USA) were employed for statistical analysis and creating the graphs. At least one representative microorganism from each genus of the microorganisms commonly causing SA and PJI was investigated. Quantitative data were presented as mean ± standard deviation (SD) or median and range, as appropriate. To determine whether sample data has a normal distribution and equal variances the Shapiro–Wilk and the Brown-Forsythe tests were used. To compare different groups the one-way ANOVA test was performed. Statistical significance was established at *p* < 0.05. The Pearson correlation coefficient was used to measures a linear correlation between D-lactate concentration and bacterial growth.

## Results

### D- and L-lactate production in planktonic microorganisms

**Figure 1:**
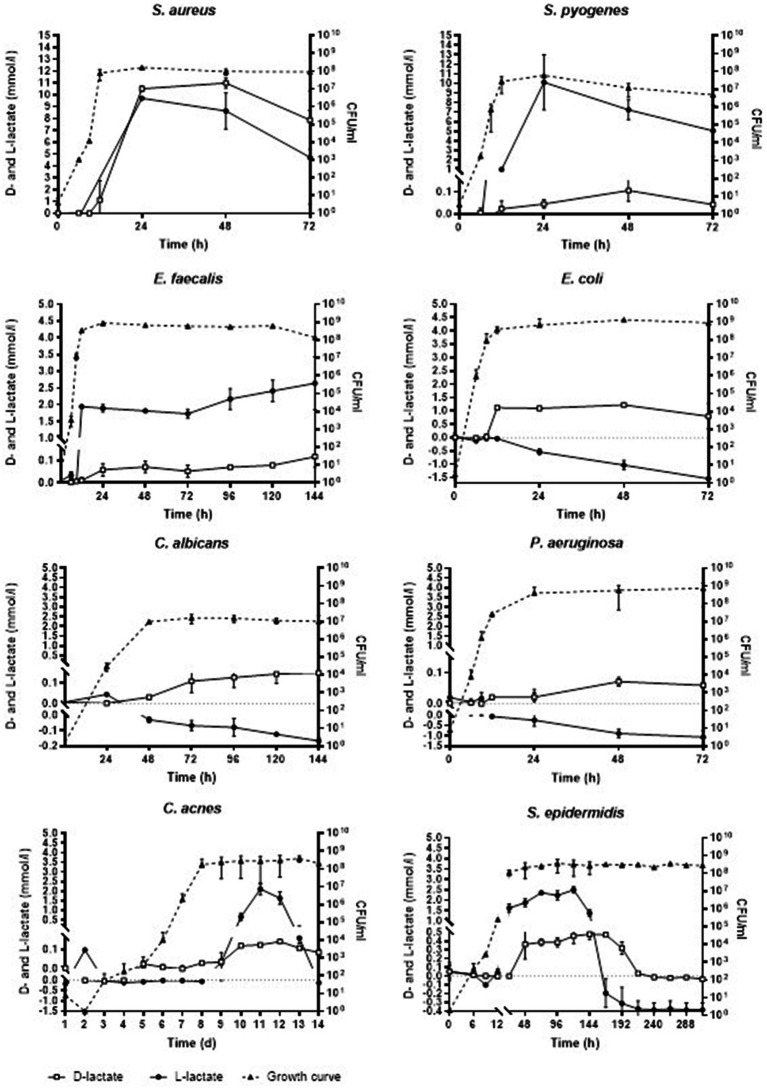
Concentration of D- and L-lactate lactate produced by investigated pathogens in planktonic form with corresponding growth curves. Time is expressed in hours (h) or days (d). Error bars represent the standard deviation (SD) of the mean. CFU/ml, colony-forming unit per milliliter.

Table 1 and Figure 1 show the mean and the standard deviation (SD) of minimal and maximal concentrations of D- and L-lactate produced by investigated pathogens in planktonic form with corresponding CFU in growth media. Production of D-lactate was observed in all tested microorganisms. *E. coli* exhibited the highest rate of D-lactate production, with minimal (0.03 mmol/L) and maximal (1.22 mmol/L) D-lactate concentrations observed at 9 and 48 h of incubation, respectively. In *S. aureus* minimal (1.13 mmol/L) and maximal (10.99 mmol/L) concentration of D-lactate was observed at 12 and 48 h of incubation, respectively, followed by *S. epidermidis* (0.26 and 0.48 mmol/L after 48 and 144 h), *S. pyogenes* (0.02 and 0.12 mmol/L at 12 and 48 h), *E. faecalis* (0.01 and 0.12 mmol/L at 12 and 144 h), *P. aeruginosa* (0.02 and 0.07 mmol/L at 12 and 48 h), *C. albicans* (0.03 and 0.15 mmol/L after 48 and 120 h) and *C. acnes* (0.03 and 0.14 mmol/L at 8 and 12 days).

**Table 1:** Minimal and maximal concentrations of D- and L-lactate produced by investigated pathogens in planktonic form with corresponding CFU/ml.

ATCC Strain	D-lactate, mmol/l (±SD; min/max)	Time, h (min/max)	CFU/ml (±SD)	L-lactate, mmol/l (±SD; min/max)	Time, h (min/max)	CFU/ml (±SD)
*S. aureus*	1.13 (±1.62)/10.99 (±0.42)	12/48	7.80E+07 (±4.80E+07)/9.56E+07 (±3.53E+07)	9.71 (±0.17)/9.71 (±0.17)	24/24	1,59E+08 (±4.68E+07)/1,59E+08 (±4.68E+07)
*S. epidermidis*	0.26 (±0.02)/0.48 (±0.06)	48/144	2.20E+08 (±1.68E+08)/2.70E+08 (±1.84E+08)	0.04 (±0.03)/2.50 (±0.14)	12/120	2.61E+05 (±7.07E+03)/3.10E+08 (±2.40E+08)
*S. pyogenes*	0.02 (±0.03)/0.12 (±0.07)	12/48	1.20E+07 (±2.12E+07)/6.00E+06 (±8.48E+06)	0.36 (±0.23)/10.12 (±2.87)	9/24	2.40E+05 (±6.78E+05)/5.40E+07 (±8.48E+06)
*E. faecalis*	0.01 (±0.01)/0.12 (±0.04)	12/144	4.10E+08 (±7.07E+07)/1.50E+08 (±1.41E+07)	1.94 (±0.03)/2.64 (±0.79)	12/144	4.10E+08 (±7.07E+07)/1.50E+08 (±1.41E+07)
*E. coli*	0.03 (±0.06)/1.22 (±0.01)	9/48	9.07E+07 (±1.26E+08)/1.32E+09 (±2.83E+08)	0	-	
*P. aeruginosa*	0.02 (±0.006)/0.07 (±0.01)	12/48	2.73E+07 (±8.08E+06)/5.73E+08 (±5.26E+08)	0	-	
*C. albicans*	0.03 (±0.006)/0.15 (±0.03)	48/144	9.70E+06 (±1.41E+05)/1.03E+07 (±1.23E+07)	0	-	
*C. acnes*	0.03 (±0.004)/0.14 (±0.004)	8/12^*^	1.72E+08 (±1.90E+08)/3.02E+08 (±2.66E+08)	0.04 (±0.04)/2.11 (±0.30)	9/11^*^	2.40E+08 (±2.11E+08)/2.67E+08 (±2.52E+08)

Lactate concentrations and CFU/ml are presented as mean (±SD). SD, standard deviation; CFU/ml, colony-forming unit per milliliter.

* Days.

No L-lactate production was detected in *E. coli*, *P. aeruginosa* and *C. albicans*. In *S. pyogenes* minimal (0.36 mmol/L) and maximal (10.12 mmol/L) L-lactate concentration was observed at 24 and 48 h, respectively, followed by *E. faecalis* (1.94 and 2.64 mmol/L at 12 and 144 h), *S. epidermidis* (0.04 and 2.5 mmol/L at 12 and 120 h), *C. acnes* (0.04 and 2.11 mmol/L at 9 and 11 days). *S. aureus* started producing L-lactate at 24 h with 9.71 mmol/L which was withal the maximal L-lactate concentration produced by this pathogen.

**Figure 2:**
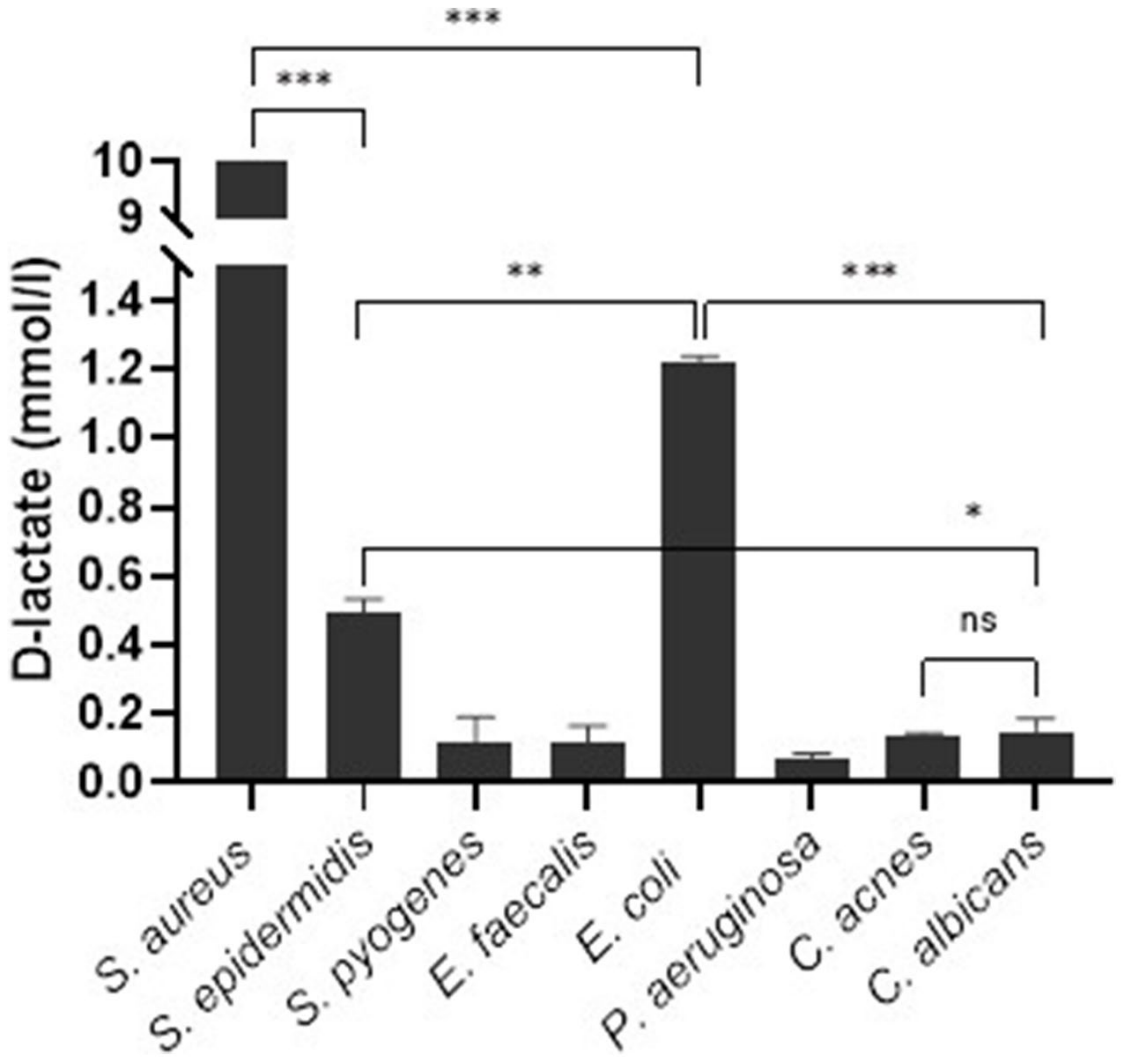
Synovial fluid D-lactate concentration stratified according to microorganism. Error bars represent the standard deviation (SD) of the mean. ^***^, *p* < 0.001; ^**^, *p* < 0.01; ^*^, *p* < 0.05; ns, not significant (*p* > 0.05).

The Shapiro–Wilk and Brown-Forsythe tests showed normal distribution of sample data and equality of means. One-way ANOVA analysis showed no significant difference in D-lactate concentration produced by *S. pyogenes, E. faecalis, P. aeruginosa*, *C. acnes,* and *C. albicans*, [*F* (4.6) = 1.615, *p* = 0.285]. The mean D-lactate concentration in *S. aureus* was significantly higher than in other bacteria as well as fungi (*p* < 0.001). *E. coli* produced significantly higher amount of D-lactate compared to *S. epidermidis* (*p* = 0.01) and other microorganisms except of *S. aureus. S. epidermidis* produced significantly higher amount of D-lactate compared to *C. albicans* (*p* < 0.05) as well as other microorganisms except of *S. aureus* and *E. coli* (Figure 2).

There was a very strong positive correlation between bacterial growth and D-lactate concentration across various microorganisms: *S. aureus* (*r* = 0.9, *p* = 0.027), *S. epidermidis* (*r* = 0.9, *p* = 0.0001), *S. pyogenes* (*r* = 0.8, *p* = 0.048), *E. faecalis* (*r* = 0.9, *p* = 0.002), *E. coli* (*r* = 0.8, *p* = 0.035), *P. aeruginosa* (*r* = 0.9, *p* = 0.005), *C. acnes* (*r* = 0.9, *p* = 0.0001), *C. albicans* (*r* = 0.8, *p* = 0.031). Similarly, there was a very strong correlation between bacterial growth and L-lactate concentration in the cultures of *S. aureus* (*r* = 0.9, *p* = 0.031), *S. epidermidis* (*r* = 0.9, *p* = 0.0001), *S. pyogenes* (*r* = 0.8, *p* = 0.044), and *E. faecalis* (*r* = 0.9, *p* = 0.001). A strong positive correlation was observed in *C. acnes* culture (*r* = 0.6, *p* = 0.036). However, a very strong negative correlation was found in the cultures of *E. coli* (*r* = −0.9, *p* = 0.014), *P. aeruginosa* (*r* = −0.9, *p* = 0.0006), and *C. albicans* (*r* = −0.7, *p* = 0.046).

### D- and L-lactate measurement in liquid culture media (control samples)

In TSB, the concentration of D- and L-lactate (mean ± SD) was 0.14 ± 0.06 mmol/L and 1.72 ± 0.40 mmol/L, respectively. In TSB + 5% DSB, the concentrations were 0.17 ± 0.03 mmol/L and 2.03 ± 0.28 mmol/L. In Saburaud broth, the concentrations were 0.09 ± 0.01 mmol/L and 0.76 ± 0.1 mmol/L.

### Influence of glucose supplementation on D-lactate production

**Figure 3:**
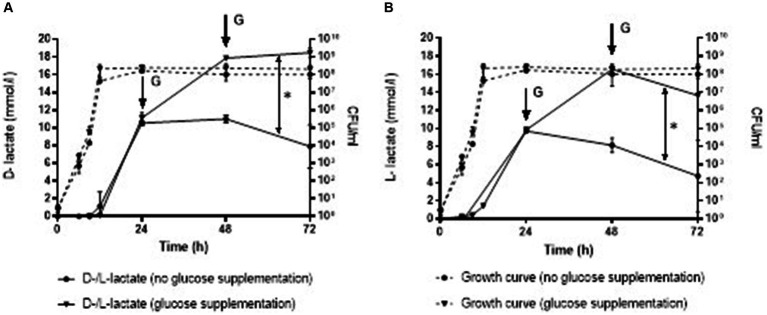
Production of D-lactate **(A)** and L-lactate **(B)** in *S. aureus* without and with glucose supplementation at 24 and 48 h. Error bars represent the standard deviation (SD) of the mean. CFU/ml, colony-forming unit per milliliter; G, glucose supplementation; ^*^, *p* < 0.05.

*Staphylococcus aureus* culture after addition of glucose at 24 and 48 h contained significantly higher D-lactate (mean ± SD, 18.48 ± 0.17 mmol/L), as well as L-lactate (13.65 ± 0.13 mmol/L) after 72 h of incubation compared to the *S. aureus* culture without glucose supplementation (D-lactate 7.90 ± 0.17 mmol/L and L-lactate 4.73 ± 0.13 mmol/L), *p* = 0.010 and *p* = 0.239, respectively (Figure 3).

### D- and L-lactate production in *Staphylococcus epidermidis* biofilm

*Staphylococcus epidermidis* biofilm in aerobic conditions started producing D-lactate at 6 h of incubation with mean (±SD) concentration of 0.02 ± 0.01 mmol/L and bacterial count of 4.50E+04 ± 7.07E+03 in the supernatants of biofilm culture (minimal production of D-lactate). At 168 h the mean concentration of D-lactate was 0.34 ± 0.01 mmol/L with bacterial count of 5.10E+08 ± 1.41E+07 CFU/ml (maximal concentration of D-lactate). Anaerobically minimal 0.05 ± 0.02 mmol/L and maximal 0.53 ± 0.003 mmol/L D-lactate concentration was observed at 9 and 240 h, respectively. Up to 144 h of incubation there was no significant difference in D-lactate concentration when bacterial biofilm was cultured either anaerobically or aerobically 0.23 ± 0.16 mmol/L (range, 0.05–0.45 mmol/L) and 0.16 ± 0.12 mmoL/L (range, 0.02–0.29 mmol), respectively, *p* = 0.247. After 144 h there was a significant decrease in D-lactate concentration in aerobic culture whereas D-lactate concentration in anaerobic culture remains stable [0.20 mmol/L ± 0.12 (decreasing from 0.34 to 0.01 mmol/L) and 0.49 mmol/L ± 0.04 (range, 0.47–0.52 mmol/L), respectively, *p* = 0.0006]. *S. epidermidis* biofilm in aerobic conditions started producing L-lactate at 6 h of incubation with mean concentration of 0.03 ± 0.02 mmol/L and bacterial count of 4.50E+04 ± 7.07E+03 CFU/ml in the supernatant of biofilm culture (minimal production of L-lactate). At 120 h the mean concentration of L-lactate was 0.45 ± 0.11 mmol/L with bacterial count of 4.04E+08 ± 2.05E+08 CFU/ml (maximal concentration of L-lactate). Anaerobically minimal 0.49 ± 0.10 mmol/L and maximal 0.49 ± 0.05 mmol/L concentration of L-lactate was observed at 9 and 144 h, respectively. The mean L-lactate concentration was similar at the first week of incubation in both aerobiosis 0.27 mmol/L ± 0.17 (range, 0.03–0.45 mmol/L) and anaerobiosis 0.26 mmoL/L ± 0.16 (range, 0.49–0.49 mmol/L), *p* = 0.700 and significantly decreased after 192 h in aerobic incubation −0.25 ± 0.03 mmol/L (decrease from −0.18 to −0.27 mmoL/L) whereas in anaerobic culture the mean concentration of L-lactate remains stable 0.17 mmol/L ± 0.04 (range, 0.15–0.21 mmol/L), *p* = 0.008.

**Figure 4:**
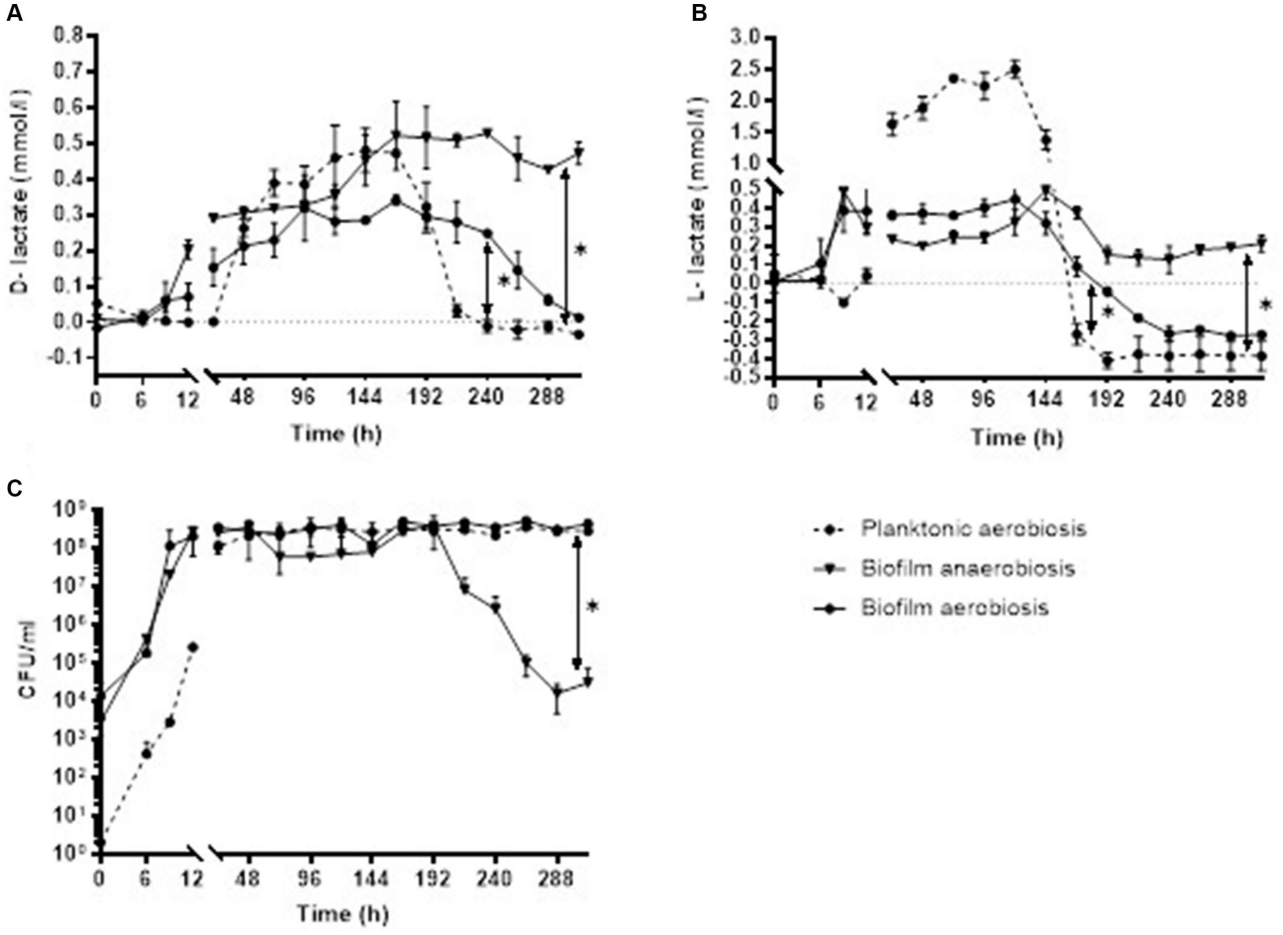
Concentration of D-lactate **(A)** and L-lactate **(B)** with corresponding CFU/ml **(C)** of *S. epidermidis* planktonic and biofilm culture in aerobic and anaerobic conditions. Error bars represent the standard deviation (SD) of the mean. CFU/ml, colony-forming unit per milliliter.

Analysis on comparison of D- and L-lactate production in planktonic and biofilm bacteria showed that at the first 8 days of incubation D-lactate concentration was similar in planktonic (0.24 mmol/L ± 0.21, range, 0.26–0.48 mmol/L) and aerobic (0.19 mmol/L ± 0.12, range, 0.02–0.34 mmol/L) as well as anaerobic (0.30 mmol/L ± 0.18, range, 0.05–0.52 mmol/L) biofilm form, *p* = 0.389 and *p* = 0.513, respectively. After 192 h of incubation the concentration of D-lactate decreased significantly in planktonic culture [−0.01 mmol/L ± 0.02 (decrease from 0.03 to −0.03 mmol/L)] compared to aerobic biofilm [0.15 mmol/L ± 0.11 (decrease from 0.28 to 0.01 mmol/L), *p* = 0.015] as well as anaerobic biofilm [0.47 mmol/L ± 0.04 (decrease from 0.53 to 0.43 mmol/L), *p* = 0.008]. Investigation of L-lactate concentration revealed similar results. At the first 7 days of incubation there was no significant difference in L-lactate production between planktonic [1.20 mmol/L ± 0.08 (range, 0.04–1.38 mmol/L)] and aerobic [0.31 mmol/L ± 0.14 (range, 0.03–0.32 mmol)] as well as anaerobic [0.25 mmol/L ± 0.16 (range, 0.19–0.49 mmol/L)] biofilm form, *p* = 0.352 and *p* = 0.239). Whereas after 7 days L-lactate decreased significantly in planktonic culture [−0.37 mmol/L ± 0.05 (decrease from −0.26 to −0.41 mmol/L)] compared to aerobic biofilm culture [−0.17 ± 0.14 (range, 0.09–0.27 mmol/L), *p* = 0.002] and anaerobic biofilm [0.19 mmol/L ± 0.08 (range, 0.13–0.37 mmol/L; *p* = 0.001; Figure 4].

## Discussion

The management of aseptic inflammation in native and prosthesis joints essentially differs from the one of microbial infection, therefore an accurate diagnosis of SA and PJI is crucial for the planning of the optimal treatment strategies (Zimmerli et al., 2004; Tande and Patel, 2014).

The “gold standard” tests in the diagnostic workup are represented by synovial fluid leukocyte count and culture. Both tests lack either sensitivity or specificity. Leukocytes may be elevated due to other inflammatory non-infectious conditions. Bacterial culture requires time until detection of microbial growth and has limited sensitivity, particularly in patients with low-grade PJI (Corvec et al., 2012; Morgenstern et al., 2018).

Therefore, a pathogen-specific biomarker which can promptly and accurately diagnose the microbial infection is an innovative approach in the management of SA and PJI.

L-lactate is already a part of clinical routine when it comes to diagnosis of infection, such as meningitis or sepsis (Sakushima et al., 2011; Hussein et al., 2017; Evans et al., 2021). Some studies have shown good performance of L-lactate test in synovial fluid for the diagnosis of PJI with sensitivity and specificity comparable to that of leukocyte count (Lenski and Scherer, 2014; Sharma et al., 2020). Lately, there has been a growing interest in D-lactate for the diagnosis of SA and PJI. Chen et al. evaluated the performance of D-lactate for diagnosing periprosthetic joint infection (PJI) investigating serum and synovial fluid samples from patients with PJI and aseptic prosthesis failure (Chen et al., 2022). The study demonstrated that in the PJI group, synovial fluid D-lactate levels were significantly higher than serum D-lactate levels. Synovial D-lactate showed a sensitivity of 96%, compared to 88% for serum D-lactate. The ability of microorganisms to form biofilms on implant surfaces and persist as local infections, rather than spreading systemically, underscores the importance of analyzing synovial fluid for accurate PJI diagnosis. Several clinical studies, utilizing synovial fluid analysis, have shown that D-lactate has superior sensitivity and specificity for diagnosing PJI when compared to leukocyte count and culture (Yermak et al., 2019; Fuchs et al., 2022). However, the ability of the most common microorganisms, causing SA and PJI, to produce two lactate stereoisomers (D- and L-lactate) has not been thoroughly investigated. It is challenging to draw conclusions from clinical studies about the intrinsic D-lactate production by different species. Due to the low performance of bacterial cultures in detecting biofilm infections, especially mixed-species infections, up to 30% of infected cases remain culture-negative (Corvec et al., 2012; Morgenstern et al., 2018). This limitation can lead to over- or underestimation of D-lactate concentrations in clinical samples when investigating intrinsic D-lactate production by different species. To address this, an *in vitro* model with controlled environments was chosen to evaluate D- and L-lactate production by the most common pathogenic microorganisms causing septic arthritis (SA) and PJI. In our study, prior to performing D- and L-lactate tests, the samples were made protein-free using 10 kDa filter. Martí et al. (1997) underlined in their study the importance of deproteinizing samples for D-lactate analysis when enzymatic methods are used in order to avoid the possible underestimation of D-lactate concentration in samples with high concentration of L-lactate, and vice versa (Martí et al., 1997).

First, the production of D- and L-lactate was investigated in planktonic bacteria grown aerobically. *S. aureus* produced the largest amount of D-lactate as well as L-lactate which was significantly higher compared to other investigated microorganisms (*p* < 0.001). The results correlate with previously published clinical studies investigating performance of D-lactate for the diagnosis of PJI. The authors found that the concentration of synovial fluid D-lactate was significantly higher in infections caused by highly virulent bacteria, such as *S. aureus,* than by coagulase-negative staphylococci, typical low virulent pathogens (Karbysheva et al., 2020b). Nevertheless, *S. epidermidis* produced the measurable amount of D- and L-lactate as it possess both stereoisomers of the enzyme lactate dehydrogenase (Stockland and San Clemente, 1969), therefore producing both enantiomers of lactate.

The second largest producer of D-lactate was *E. coli.* The concentration of D-lactate produced by this high virulent pathogen was significantly higher compared to other tested microorganisms, except of *S. aureus* (*p* < 0.01). This pathogen started to produce measurable amount of D-lactate much faster than other tested strains, which could be attributed to its higher growth rate, compared to other investigated bacteria and fungi. In contrast to D-lactate, the production of L-lactate was not observed in this microorganism. Moreover, the concentration of L-lactate, naturally present in liquid media, decreased during the bacterial growth. This is explained by the fact that intrinsically *E. coli* does not produce L-lactate in noticeable amounts, but rather utilizes it as a carbon source via a dehydrogenation reaction mediated by an FMN-dependent L-lactate dehydrogenase (Niu et al., 2014).

Similar findings were observed in *P. aeruginosa.* It produced D-lactate, but not L-lactate. Lin et al. (2018) described four different lactate dehydrogenases of *P. aeruginosa* that oxidate D- and L-lactate to pyruvate, or reduce pyruvate to D-lactate, but no L-lactate dehydrogenase with ability to reduce pyruvate to L-lactate was observed, which explains the lack of L-lactate production (Lin et al., 2018).

*Candida albicans* produces D-lactate mostly using enzymes glyoxalases to catabolize methylglyoxal, a product of a metabolic pathway of glycolysis (Hasim et al., 2014). Furthermore, *C. albicans* can utilize lactate as an alternative carbon source but its mechanism is unclear, as well as the fact which stereoisomer of lactate is utilized more (Sun et al., 2023). In our study we have detected the production of D-lactate in *C. albicans*, but not L-lactate. Similar to *E. coli* and *P. aeruginosa* culture, L-lactate concentration originating from liquid media decreased during *C. albicans* cultivation, confirming that *C. albicans* mainly utilizes L-lactate and produces D-lactate. Concentration of D-lactate produced by *C. albicans* was similar to that produced by *S. pyogenes, E. faecalis, P. aeruginosa*, *and C. acnes* (*p* > 0.05) and significantly less compared to *S. aureus, S. epidermidis*, *and E. coli* (*p* < 0.01). Microbiological diagnosis of fungal infections represents a challenge due to the relatively low sensitivity and long cultivation time (Fang et al., 2023). These limitations emphasize the necessity and importance of non-culture diagnostic tools with faster turnaround time and higher performance for diagnosing fungal infections. Utilizing a microbial biomarker that also detects fungal infection is a great interest in the management of SA and PJI.

Infection caused by *C. acnes* poses a challenge for the diagnosis, as this is a slow growing, fastidious pathogen, requiring up to 14 days of cultivation (Prinz et al., 2022). Due to its low virulence, it often causes a mild host reaction and presents with falsely low number of leukocytes in synovial fluid (Renz et al., 2018; Izakovicova et al., 2019; Jeverica et al., 2020), therefore going unnoticed and mistaken for aseptic loosening of an implant. In our study, for the first time D-lactate and L-lactate production was described in *C. acnes* and showed measurable concentrations of both stereoisomers.

Irrespective of the microorganisms, we found a very strong positive correlation between bacterial growth and D-lactate concentration in the growth media. In contrast, L-lactate positively correlated with bacterial growth only in the cultures of *S. aureus, S. epidermidis, S. pyogenes, E. faecalis, and C. acnes*. Interestingly, L-lactate showed a very strong negative correlation with bacterial growth in the cultures of *E. coli, P. aeruginosa, and C. albicans*. Smith et al. (1986) reported the production of D- and L-lactate in measurable quantities by frequently encountered human bacterial pathogens under anaerobic growth. In different species the concentration of D-lactate varied from 0.4 to 34 mM and L-lactate from 1.2 to 23 mM (Smith et al., 1986). In the present study we found concordant results in production of D-lactate and discordant in L-lactate. While D-lactate was detected in all tested microorganisms in measurable concentrations, the production of L-lactate was absent in *E. coli*, *P. aeruginosa*, and *C. albicans.* The reason for discordant results might be due to different growth conditions, culture media or different test kits for D-lactate detection. On the other side (Wellmer et al., 2001) have tested *S. aureus* and *E. coli* in a setup similar to one in our study using TSB as a growth media and enzymatic method for lactate detection. The results were comparable to our study indicating that *S. aureus* produced both enantiomers of lactate, while *E. coli* almost exclusively produced D-lactate (97.2% of total lactate; Wellmer et al., 2001).

In our study the presence of the glucose, as the best fermentation fuel, in the culture media on production of D-lactate was investigated. Addition of glucose to *S. aureus* culture led to an immediate increase in the production of D-lactate. Similar results were reported by Smith et al. (1986) the authors found that the accumulation of D-lactate increased dramatically as glucose was replenished every 6 h in the liquid medium with *E. coli* (Smith et al., 1986). These findings suggest that in *in vivo* situation, with the constant blood perfusion to the infection site, even higher concentrations of D-lactate can be expected.

To investigate the D- and L-lactate production in biofilm bacteria *S. epidermidis* was chosen for biofilm formation as it is the most common pathogen causing PJI and known to be good biofilm former (Karbysheva et al., 2020a). The natural habitat of *S. epidermidis* are the hypoxic environments, which are frequently encountered at or near artificial devices within the host organism (Oliveira et al., 2023). There this microaerophilic bacterium reacts manufacturing proteins and polysaccharides that enable them attaching to surfaces and forming biofilms as barriers against antibiotics and other toxic substances (Uribe-Alvarez et al., 2015; Pedroza-Dávila et al., 2020). In our study the analysis of the medium, covering the biofilms, showed a significant reduction in CFU count when bacterial biofilms were cultivated anaerobically compared to aerobic cultivation. Reports indicate that when oxygen levels are low, staphylococci prioritize the production of biofilm matrix components over cell division. Conversely, the biofilm generation decreased as the oxygen concentration increased (Uribe-Alvarez et al., 2015; Pedroza-Dávila et al., 2020).

Analysis of the supernatants of biofilms showed that the D- and L-lactate concentration produced by *S. epidermidis* biofilm, grown in anaerobiosis and aerobiosis, was similar at the first week of incubation (D-lactate, *p* = 0.239; L-lactate, *p* = 0.700) and significantly decreased for both enantiomers after 7 days in aerobic conditions (D-lactate, *p* = 0.0006; L-lactate, *p* = 0.008). Pedroza-Dávila et al. (2020) reported that in *S. epidermidis*, elevating oxygen concentration led to an increase in the rate of oxygen consumption, while fermentation was inhibited (Pedroza-Dávila et al., 2020). Additionally, in bacterial cultures lactate is utilized as a carbon source after glucose is depleted from the medium (Oliveira et al., 2023). Comparing D-lactate and L-lactate production in planktonic and biofilm bacteria we found that during the first week of incubation D-lactate concentration was similar in planktonic and biofilm form. After 192 h of incubation the concentration of D-lactate decreased significantly in planktonic culture compared to aerobic biofilm (*p* = 0.015) as well as anaerobic biofilm (*p* = 0.008). Investigation of L-lactate concentration revealed similar results: after 168 h L-lactate decreased significantly in planktonic culture compared to aerobic biofilm (*p* = 0.002) and anaerobic biofilm (*p* = 0.001). Oliveira et al. (2023) reported that *S. epidermidis* strains grown planktonically in the same medium used for the biofilms did not exhibit higher lactate levels, suggesting that the increased lactate production is specific to biofilm formation. This suggests that in patients with peri-implant infections, the biofilm form of bacteria is likely the primary source of D-lactate. Consequently, it is reasonable to anticipate a significantly higher concentration of D-lactate in *in vivo* situations.

In this study bacterial biofilms were cultivated under static conditions without addition of special supplements, potentially restricting the metabolic activity of microorganisms. Further studies utilizing flow cell technology, which enables continuous introduction of nutrients, could provide an additional information on lactate production. Nevertheless, it is essential to use methods that take into account sample dilution when calculating lactate concentration.

In summary, our study has demonstrated that the most common pathogenic microorganisms causing SA and PJI have the capability to generate measurable amounts of D-lactate in both planktonic and biofilm form, highlighting the practical value of this biomarker as an indicator for bacterial and fungal infections. In contrast to D-lactate, the absence of L-lactate production in certain tested bacteria, as well as in fungi, suggests that L-lactate is not eligible as a biomarker for diagnosing microbial infections.

## Data availability statement

The raw data supporting the conclusions of this article will be made available by the authors, without undue reservation.

## Author contributions

PM: Data curation, Formal analysis, Investigation, Methodology, Validation, Writing – original draft. MG: Conceptualization, Data curation, Formal analysis, Methodology, Writing – review & editing. AT: Conceptualization, Data curation, Project administration, Resources, Supervision, Visualization, Writing – review & editing. SK: Conceptualization, Data curation, Formal analysis, Methodology, Project administration, Supervision, Validation, Visualization, Writing – original draft, Writing – review & editing.
